# From Trends to Insights: Management of Periprosthetic Joint Infections in the Past Decade in The Netherlands

**DOI:** 10.3390/microorganisms14071458

**Published:** 2026-07-02

**Authors:** Floor C. A. Koelewijn, Jesse W. P. Kuiper, Ruben Scholten, Jakob van Oldenrijk, Matthijs P. Somford, Duncan E. Meuffels, Ewout S. Veltman

**Affiliations:** 1 Department of Orthopedics and Sports Medicine, Erasmus Medical Center, 3015 GD Rotterdam, The Netherlands; j.vanoldenrijk@erasmusmc.nl (J.v.O.);; 2 Department of Orthopaedic Surgery, Martini Ziekenhuis, 9728 NT Groningen, The Netherlands; jesse.kuiper@mzh.nl; 3 Department of Orthopaedic Surgery, Rijnstate Ziekenhuis, 6815 AD Arnhem, The Netherlands; rscholten@rijnstate.nl (R.S.); msomford@rijnstate.nl (M.P.S.)

**Keywords:** antibiotics, total hip arthroplasty, total knee arthroplasty, periprosthetic joint infection

## Abstract

The prevention and optimal treatment of periprosthetic joint infections (PJI), including antibiotic prophylaxis and DAIR (debridement, antibiotics, and implant retention), is a controversial topic. This study evaluated treatment trends over the past 10 years in the Netherlands. In November 2024, all Dutch institutions performing total hip or knee arthroplasties were contacted to complete a 21-question survey on the availability of a protocol, antibiotic prophylaxis, and treatment of PJIs. Results were compared with previously performed surveys (2013, 2016 and 2019) to describe trends in the past decade of PJI prevention and treatment. The survey response rate was 100%. The use of single-dose antibiotic prophylaxis increased from 10% in 2016 to 49% in 2024. An acute PJI protocol was present in 72% of the institutions in 2016 and 95% in 2024. During DAIR procedures, mobile components were exchanged in 94% of the institutions, whereas in 2013 this reportedly happened in only 41%. In 77% of the institutions, a periodic multidisciplinary infection meeting is implemented, representing a 22% increase since 2013. These findings demonstrate a clear tendency towards standardization and optimization of the treatment of periprosthetic infections in the Netherlands over the past decade.

## 1. Introduction

Total hip arthroplasty (THA) and total knee arthroplasty (TKA) have proven to be highly effective in treating end-stage osteoarthritis, improving pain relief and functional outcomes [1]. In the Netherlands, approximately 37.000 THAs and 28.000 TKAs are performed annually [2]. Despite their high success rates, periprosthetic joint infection (PJI) is one of the most devastating complications, affecting 1–2% of the patients [3]. Over the last 5–10 years, periprosthetic joint infection has become the most frequent reason for revision arthroplasty in the Netherlands [4]. PJI leads to prolonged treatment, functional impairment, and substantial healthcare costs [5,6]. As the number of primary arthroplasties continues to rise annually, the absolute number of PJI is expected to increase accordingly.

To minimize the risk of PJI, antibiotic prophylaxis regimens are widely used [7]. Based on extensive research, local guidelines have been formulated which leave little controversy regarding the recommended type and timing of antibiotics (a cephalosporin such as cefazolin, 15–60 min before incision) [8]. In addition, the Dutch Orthopaedic Infection society (WOI) was established by the Dutch National Orthopaedic Association (NOV) to promote centralization and harmonization for PJI patients in the Netherlands. This has led to a nationwide 2015 recommendation on the treatment of PJIs [9]. In the case of suspected early PJI, debridement, antibiotics, and implant retention (DAIR) is usually indicated [10]. After performing DAIR, empiric antibiotics are administered while awaiting intraoperative tissue cultures. However, the 2015 guideline does not specify which empiric antibiotic agent to use, leaving the choice and duration of empiric therapy to local protocols [9].

Previous independent survey studies in the Netherlands from 2013, 2016 and 2019 showed that not all institutions had a set protocol for suspected PJI and that, for example, modular parts were not always exchanged during a DAIR procedure [11,12,13]. Furthermore, a large variation in empiric antibiotics in the case of suspected PJI was described [11,13]. The introduction of new national guidelines may have changed daily practice, yet it remains unknown how well these are currently implemented [8,9].

The aim of this study was to evaluate the use of standardized protocols on systemic antibiotic prophylaxis, surgical treatment and empiric antibiotic treatment in suspected PJI in the Netherlands in 2024. Secondly, results were compared with the surveys conducted in 2013, 2016 and 2019 in the Netherlands to analyze trends in treatment approaches over the past decade, as possibly driven by the implementation of new national guidelines.

## 2. Materials and Methods

In November 2024, a twenty-one-question online survey, regarding the availability of a protocol, antibiotic prophylaxis and treatment of periprosthetic infections was constructed and sent via Google Forms (cloud-based version, Google, Mountain View, CA, USA) to all orthopaedic departments performing THA and/or TKA in the Netherlands. No exclusion based on number of annually procedures performed or years of experience was made. The questionnaire was completed by one board-certified orthopaedic surgeon per institution. The selected orthopaedic surgeon was specialized in either total hip arthroplasty or total knee arthroplasty, or both, preferably with expertise in PJI surgery. If no surgeon or multiple surgeons with expertise in PJI surgery were present at the institution, one surgeon was randomly selected to complete the survey. Individual orthopaedic surgeons of non-responding departments were contacted digitally or by phone to increase the response rate. Residents were not approached to complete the survey. All survey responses were collected within one month. Survey responses were not collected anonymously for the researchers. Any ambiguities in survey responses were clarified, if necessary, by contacting the selected orthopaedic surgeon. Topics were divided into primary arthroplasty, revision arthroplasty, suspected acute infection and PJI protocol. The questions were primarily focused on the initial treatment of PJI, rather than on treatment failure or antibiotic resistance. An English translation of the survey can be found in Table 1.

Relevant data from previous, non-identical surveys in 2013, 2016 and 2019 were derived to describe trends over the past decade within the Netherlands. Data management and analysis was performed using Microsoft Excel 365 software, version 2501 (Microsoft Corporation, Redmond, WA, USA). Survey responses were described using frequencies and percentages.

## 3. Results

All hospitals and clinics performing THA and TKA in the Netherlands in 2024, according to the Dutch Arthroplasty Register (LROI), responded to the survey. A total of 79 institutions were included, representing a response rate of 100%. In total, 7 academic hospitals (9%), 63 regional hospitals (80%) and 9 private orthopaedic clinics (11%) were included. When different sites of the same institution followed the same protocol, they were considered a single institution. All individual survey responses were complete.

### 3.1. Primary Arthroplasty

A single dose of antibiotic prophylaxis was administered in 39 (49%) institutions, while another 39 (49%) institutions administered multiple doses (defined as ≥2 doses) within 24 h. In one (1%) institution, antibiotic prophylaxis was dependent on the ASA (American Society of Anesthesiologists) score (ASA 1–2 single-dose, ASA 3–4 multiple dose). In eight of the nine private clinics, single-dose antibiotic prophylaxis was administered. In the academic and regional hospitals, single-dose and multiple-dose regimens were equally distributed.

As prophylaxis, cefazolin is prescribed in 77 institutions (97%), cefuroxime in 1 (1%), and a single dose of cefazolin preoperatively followed by cefuroxime postoperatively in 1 (1%). This is in line with the Dutch Orthopaedic Association (NOV) guideline, which recommends the use of a cephalosporin as prophylactic antibiotic [14].

**Figure 1:**
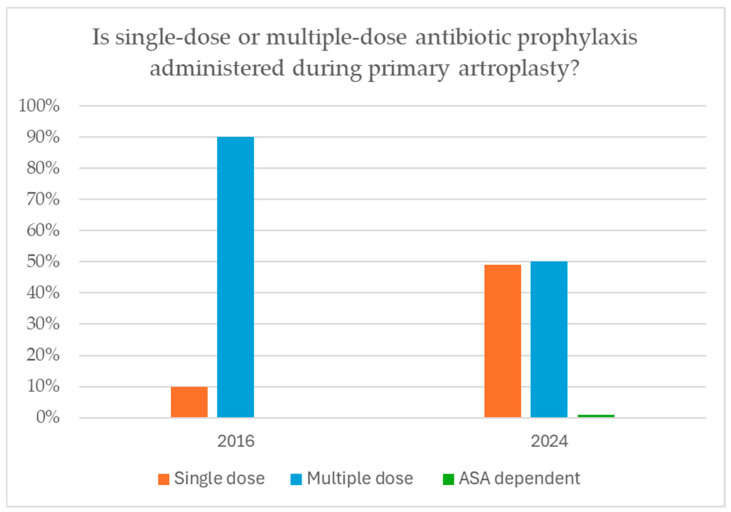
Antibiotic prophylaxis in primary arthroplasty.

Compared to 2016, single-dose antibiotic prophylaxis use increased from 10% to 49% (Figure 1).

### 3.2. Revision Arthroplasty

For revisions, single-dose antibiotic prophylaxis was administered in 28 (36%) of the institutions, while 39 (51%) administered multiple doses within 24 h. In the remaining institutions, multiple-dose antibiotic prophylaxis was administered for more than 24 h (one institution for 48 h, one for 72 h, one for 5 days, and seven institutions for multiple days until culture results were known). Two private orthopaedic institutions did not perform revision arthroplasty. Cefazolin was prescribed in 72 (94%) institutions, cefuroxime in 2 (3%), cefuroxime + ciprofloxacin in 1 (1%), vancomycin + ciprofloxacin in 1 (1%) and in 1 (1%) institution 24 h of cefazolin was prescribed, followed by cefuroxime until culture results were known.

Antibiotic prophylaxis was administered at the start of surgery in 57 (74%) institutions and after tissue cultures were taken in 19 (25%) institutions. In one (1%) institution, administration depended on surgical indication. All private orthopaedic clinics administered antibiotic prophylaxis at the start of surgery. No relevant differences were observed between academic and regional hospitals.

No relevant data on this topic was described in the previous survey studies.

### 3.3. Suspected Acute PJI

**Figure 2:**
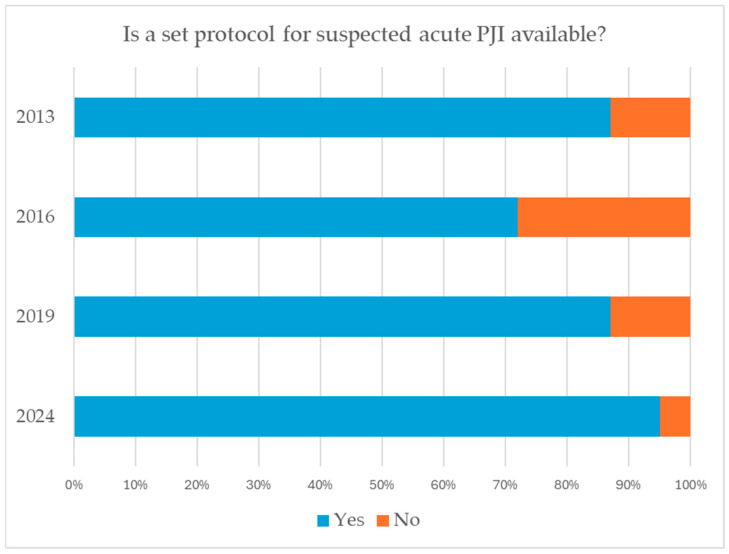
Availability of a set protocol for antibiotic treatment in suspected acute PJI.

The availability of a set protocol for antibiotic treatment in the case of suspected acute postoperative PJI increased over the past decade. In 2024, a set protocol was available in 75 (95%) of the institutions. Two institutions reported referring all suspected acute PJI elsewhere. In 2013, 2016 and 2019, a set protocol was reported in respectively 87%, 72%, and 87% of the institutions (Figure 2).

The timeframe in which institutions considered DAIR a viable option for suspected acute PJI varied substantially: 10 institutions (13%) reported a maximum of 4 weeks, 25 institutions (32%) up to 6 weeks, 11 institutions (14%) up to 8 weeks, and 25 institutions (32%) up to 12 weeks. In the remaining institutions, the decision was either case-dependent (5%) or determined in consultation with an academic center (3%). Empiric antibiotic treatment after DAIR consisted of a combination of agents in 41 (55%) of the institutions and monotherapy in 33 (45%). There was a large variation in the type of prescribed antibiotics (Table 2). In 2019, 72% of the institutions used monotherapy and 28% combination therapy, implying a trend towards combination therapy in 2024.

**Figure 3:**
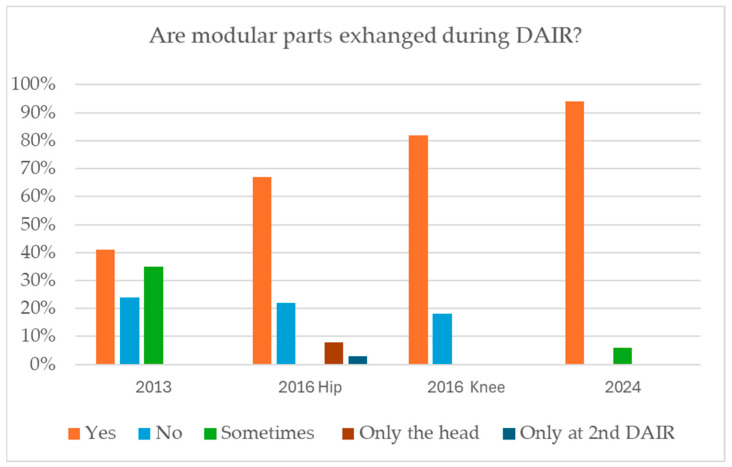
The exchange of modular components over the course of time.

In 72 (94%) of the institutions, modular components were always exchanged during a DAIR procedure. In the five remaining institutions, it was dependent on the surgeon, joint, or surgical approach. The change in exchange of modular components over the course of time can be seen in Figure 3. DAIR procedures were registered in the LROI registry in 98% of the institutions, of which 45% indicated that DAIR procedures were not reported in rare cases when modular components could not be exchanged. One institution reported never registering DAIR procedures. In 2016, 64% of the DAIR procedures were registered in the LROI.

### 3.4. PJI Protocol

A regular multidisciplinary PJI meeting was standard care in 55% of the hospitals treating PJI in 2013, increasing to 77% in 2024. Among the respondents in 2024, 62% held meetings weekly, 7% every two weeks, 7% monthly, and 2% every three months. The orthopaedic surgeon and microbiologist were standard participants in the meeting. In 62% and 21% of the institutions, respectively, the infectious disease specialist and pharmacist were also involved. Other specialists were less frequently present (16% trauma surgeon, 7% geriatrician, 2% anesthesiologist/nurses/social work). All seven academic institutions held weekly meetings, while the four private orthopaedic clinics treating PJI only held meetings on indication.

In 58 (89%) of the institutions treating suspected chronic PJI, a set diagnostic protocol was available. Fourteen institutions reported referring all suspected chronic PJI to other centers.

Laboratory tests and synovial fluid aspiration for culture were performed in all institutions who treat chronic infections. Synovial leukocyte count and synovial calprotectin were performed in 71% and 14% of the institutions, respectively. Imaging techniques, such as SPECT, PET scan or CT scan, were performed in respectively 39%, 21% and 15%. Less commonly used techniques included alpha defensin (8%), leukocyte esterase detection (3%), and serum-to-synovial glucose ratio (1%).

In the treatment of chronic PJI, 40 institutions (87%) performed a one-stage procedure in selected cases, while 5 (11%) consistently used a two-stage procedure in every case. When two-stage hip revision was performed, 27 (64%) always used an antibiotic-loaded interval spacer, of which prefabricated spacers were used most frequently (48%). *Girdlestone* procedures without spacers were preferred in 11 (26%) institutions, and functional spacers using regularly used prosthesis components in 5 institutions (12%). In two-stage knee revision, cement molds were the most commonly used type of spacer, applied in 32 (78%) institutions. An antibiotic holiday (defined as at least two weeks without antibiotic treatment) prior to the second procedure in two-stage revision of the hip or knee was considered standard practice in 3 (7%) of the institutions, while in 11 (26%) institutions such a holiday was never used. In 28 (67%) institutions, it was used depending on factors such as patient characteristics and bacterial resistance (Table 3). No relevant data on this topic was described in the previous survey studies.

Antibiotic treatment was continued for 12 weeks in the majority of institutions after DAIR (90%), one-stage (76%) and two-stage revision (49%). In the remaining institutions, shorter durations (e.g., 6 weeks) or individualized approaches based on cultures and clinical course were used (Table 4). No relevant data on this topic was described in the previous survey studies.

In 52 (68%) of the institutions, patients received a minimum of two weeks of intravenous (IV) antibiotic treatment. Shorter IV durations, ranging from 1 to 14 days, were used less frequently (1–3 days (4%), 3–7 days (14%), 7–14 days (6%), 10–14 days (1%)). In the remaining five (6%) institutions, IV treatment duration was dependent on causative pathogen, microbiologist recommendations, and clinical course.

A large variation in standard follow-up protocols for PJI patients was observed. In 28 (36%) institutions, patients were followed for less than one year. In 40 (52%) institutions, follow-up continued up to one year, while in 9 (12%) institutions follow-up was continued up to two years after the initial PJI treatment.

## 4. Discussion

This study shows a shift towards single-dose antibiotic prophylaxis in primary arthroplasty, with all institutions using cephalosporins. This is in line with the national guideline, which recommends a single dose of cefazolin 15 to 60 min before incision [14]. These findings are supported by a large observational study conducted by Veltman et al., which showed that risk of complete revision was comparable for single-dose and multiple-dose antibiotics [15].

In revision arthroplasty, 25% of the institutions only administered antibiotic prophylaxis after perioperative tissue cultures were taken. Several studies have shown that preoperative antibiotic prophylaxis does not influence culture results after cultures, and suggest a higher PJI rate when prophylaxis is withheld until after cultures [16,17,18]. Delaying antibiotic administration is therefore not desirable, as it may increase the risk of infection without providing significant diagnostic benefit. Interestingly, despite the literature consistently proving otherwise, the Dutch orthopaedic guideline on treatment of PJI (updated lastly in 2015) still recommends withholding antibiotic prophylaxis until cultures are taken [9]. In contrast, the recently published guideline on antibiotic treatment for PJI does recommend administration of antibiotic prophylaxis prior to surgery [8].

While laboratory tests and synovial fluid aspiration for culture were used in all institutions to diagnose PJI, histopathological tests or the involvement of a pathologist in multidisciplinary meetings were notably not mentioned. Although positive histology is an established diagnostic criterion for PJI according to the European Bone and Joint Infection Society (EBJIS), it does not appear to be a part of standard diagnostics for PJI in the Netherlands [19].

The timeframe in which institutions considered a DAIR procedure as a viable option for suspected acute PJI varied substantially from 4 up to 12 weeks. Once a DAIR procedure is no longer considered viable, the infection is typically classified as chronic PJI and treated with either one-stage or two-stage revision strategies. During two-stage revision surgery of the hip, the majority of institutions reported using a prefabricated spacer. Surprisingly, 26% of institutions reported not using a spacer between stages (Girdlestone). The use of an interval spacer has been advocated for years, and is recommended by the International Consensus Meeting (ICM) on PJI [20]. At this point, only five institutions indicated using a functional spacer consisting of regularly used prosthesis components, which is advised by the ICM. To limit the heterogeneity and to optimize outcome of surgical PJI treatments in the Netherlands, a new, updated national guideline is essential.

Over the past decade, nearly all institutions (95%) developed a set protocol for empiric antibiotic treatment following DAIR, pending the results of perioperative cultures. Remarkably, the availability of the protocol varied from 87% in 2013 to 72% in 2016, and back to 87% in 2019. This fluctuation may be explained by hospital mergers, the establishment of new institutions over time, and differences in study designs across the survey studies, including varying response rates and inclusion or exclusion of private clinics. Furthermore, 94% of the institutions reported to always exchange modular components during a DAIR procedure, which is an improvement of 53% since 2013. These findings suggest improved protocolized care in the treatment of suspected acute PJI. However, DAIR procedures were only consistently registered at the LROI database in 53% of the institutions in 2024. This is in line with a large Dutch validation study by Kamp et al., which found that 51% of the acute PJI cases were not registered due to administrative origin or DAIR procedures without component exchange [21]. This inevitably leads to an underestimation of the number of acute PJI cases in the Netherlands when looking at LROI data.

A large variation in empiric antibiotics used after DAIR in suspected acute PJI was observed (Table 1). In 2024, a new guideline was published by the Dutch Working Party on Antibiotic Policy (SWAB), which advises using vancomycin plus ceftriaxone intravenously [8]. Based on local epidemiology (e.g., high prevalence of *Pseudomonas*), vancomycin plus ceftazidime can be chosen as a second option. The Northern Infection Network Joint Arthroplasty (NINJA) guideline, followed by some hospitals in the northern part of the Netherlands, also recommends this [22]. However, we observed that only 13% of the Dutch institutions followed this advice, while cephalosporin monotherapy was much more commonly used with 37% (Table 2). This large variation may be explained by the absence of uniform national guidelines regarding empiric antibiotic treatment, as institutions primarily followed their own protocols. Although cephalosporins are sufficient for most causative organisms of PJI, such as *Staphylococcus aureus* and coagulase-negative *Staphylococci* (CNS), they are less effective against resistant CNS, Gram-negative *Bacilli* (GNB) and other biofilm-forming microorganisms, compared to vancomycin–ceftriaxone combination therapy [4,23]. The recently published guideline for antibiotic treatment of periprosthetic joint infections may improve consistency of treatment between institutions and minimize the risk of suboptimal antibiotic PJI treatment, when implemented in local protocols [8].

In 90% of the institutions, patients received 12 weeks of antibiotic treatment after a DAIR procedure, which is in line with national and international guidelines [8,20,22]. Although limited research exists, the DATIPO (Duration of Antibiotic Treatment in Prosthetic Joint Infection) study showed a trend towards a higher failure rate for patients treated with 6 weeks antibiotics compared to 12 weeks [24]. A shorter period of antibiotics may be justified in a selected group of patients, but evidence is lacking. In the majority (67%) of the institutions, patients were treated for a minimum of two weeks of IV antibiotics after surgery. Shorter IV treatment was used in 27% of the institutions. The new SWAB guideline, published in December 2024, now recommends 1 to 2 weeks of IV antibiotics, which may prompt more institutions to adopt shorter IV treatment durations [8]. In contrast, the Infectious Diseases Society of America (IDSA) guideline recommends 2–6 weeks of pathogen-specific intravenous antibiotic therapy [25]. The OVIVA (Oral versus Intravenous Antibiotics for Bone and Joint Infection) trial demonstrated non-inferiority of oral antibiotics compared to intravenous antibiotics in treatment of bone and joint infection [26]. However, PJI was grossly underrepresented, as the majority of the patients had chronic osteomyelitis or other orthopaedic infections than PJI. Other smaller studies regarding this topic have shown comparable results, yet with insufficient power or heterogenous populations [27,28]. Further research dedicated to PJI is needed to evaluate the safety and efficacy of shorter IV treatment periods.

The call for centralization and a closer multidisciplinary approach for PJI patients has been present in both Europe and North America for years. Tertreault et al. (2017) found that 55% of the patients evaluated for PJI before referral to a tertiary care center did not have a complete workup according to the American Academy of Orthopaedic Surgeons (AAOS) guidelines [29]. Improved guideline awareness and closer multidisciplinary approaches could improve PJI treatment outcomes. The French Health Ministry established a nationwide network of 24 centers for bone and joint infections (BJI) and concluded after more than 5 years of experience that this network improved infection control and promoted education and research [30]. A similarly high-volume PJI referral center was launched in the United States in 2019 and found significantly higher failure rates in patients referred >90 days after diagnosis of chronic PJI compared to patients referred within 90 days, underscoring the added value of centralization of PJI care [31].

Our study provides an overview of currently used treatment protocols and regimens in the Netherlands and treatment trends over the past decade with a 100% response rate. However, several limitations should be noted. The survey is only completed by one orthopaedic surgeon per institution, while treatment preferences may vary between surgeons within the same institution, introducing potential selection and information bias. If a protocol exists, but the contacted orthopaedic surgeon is unaware, this is reflected in our results by introducing information bias. However, as 95% of the contacted orthopaedic surgeons reported to have a protocol, the impact on our findings is expected to be small. This survey presents a snapshot of the treatment protocol at that point in time alone. Changes in protocol over time are not part of each survey on its own, but are reflected in the described trends. In addition, the questionnaire was limited to 21 questions and mainly focused on protocols and guideline implantation. Several relevant aspects, including the treatment of patients with antibiotic allergies (e.g., penicillin allergy), local microbiological resistance patterns, and institution-specific antibiograms, were not included in the survey. For this survey, only orthopaedic surgeons were contacted about their local protocol; microbiologists or infectious disease specialists were not contacted for this survey, which may limit its reliability if a separate protocol for PJI treatment would be present within one institution. Furthermore, the previous survey studies used partly different questions, making it impossible to identify trends on all topics. In addition, the 2013 survey had a response rate of only 54% compared to full participation in the other surveys, limiting the generalizability of its findings. This is further affected by the exclusion of private clinics in the 2019 survey and substantial changes in the Dutch healthcare landscape (e.g., merging of institutions). However, to our knowledge, this is the first study that provides a comprehensive review of treatment trends over time in PJI management, and provides an example of how implementation of a treatment recommendation can lead to decreased heterogeneity of treatment for PJI.

## 5. Conclusions

Over the past decade, the availability of protocols has significantly improved, the exchange of mobile components in DAIR procedures has increased and a multidisciplinary approach to treating PJI has been almost universally adopted. These trends show a tendency towards standardization and optimization in PJI management in the Netherlands, possibly driven by the recommendation for treatment of prosthetic joint infections as published by the Dutch National Orthopaedic Association (NOV) on behalf of the Dutch Orthopaedic Infection society in 2015 [9]. Our findings show that introducing a national treatment recommendation leads to improved standard of care for patients with PJI. We suggest every national orthopaedic association to introduce a specialized PJI committee and implement an evidence-based treatment recommendation to increase the standard of care in their country. The current variation in empiric antibiotic treatment regimens and surgical strategies in chronic PJI may be optimized when the recently updated guideline on PJI treatment is implemented locally [8]. In addition, further research is necessary to determine the optimal duration of intravenous antibiotics in PJI treatment.

## Figures and Tables

**Table 1:** English translation of the survey questions.

Primary arthroplasty
Do you use single- of multiple-dose antibiotic prophylaxis?
2. Which antibiotic do you use (cefazolin, cefuroxime, other)?
Revision arthroplasty
3. Do you use single- or multiple-dose antibiotic prophylaxis?
4. Which antibiotic do you use?
5. At what time do you administer prophylaxis?
Suspected acute infection
6. Do you have a set protocol for antibiotic treatment for suspected acute postoperative infections?
7. Within what timeframe a DAIR is carried out?
8. Does empiric antibiotic therapy after DAIR (in a non-septic patient) due to suspected early PJI consist of mono- or combination therapy?
9. Which empiric antibiotics are started after DAIR due to suspected early PJI (in a non-septic patient)?
10. Do you exchange modular components during a DAIR?
11. Is the DAIR registered at the LROI?
PJI protocol
12. Do you have a regular PJI meeting? Who attends?
13. Do you have a set diagnostic protocol for suspected chronic infections? What is included?
14. Which treatment algorithm do you use for chronic infections?
15. In two-stage revision of the hip, do you use a spacer?
16. In two-stage revision of the knee, do you use a spacer?
17. In case of a two-stage revision, is an antibiotic holiday planned?
18. How long are patients treated with antibiotics after DAIR?
19. How long are patients treated with antibiotics after PJI revision (DAIR/one-stage/two-stage)?
20. How long are patients treated with IV antibiotics after PJI (revision) surgery?
21. Do you have standard follow up moments?

DAIR: debridement, antibiotics, and implant retention; PJI: periprosthetic joint infection; LROI: Dutch Arthroplasty Registry.

**Table 2:** Empiric antibiotic treatment following DAIR procedure (in percentages).

	2013 (%)	2019 (%)	2024 (%)
Monotherapy			
Amoxicillin/clavulanate	8	10	6
Cephalosporin (cefazolin/cefuroxime)	32 (14/18)	38 (19/19)	37 (14/23)
Ceftriaxone	4	0	6
Clindamycin	4	1	0
Ciprofloxacin	2	0	0
Flucloxacillin	28	21	13
Vancomycin	6	1	5
Case-dependent	0	1	0
Combination therapy			
Cefuroxime/ceftriaxone	0		1
Cefuroxime/ciprofloxacin	0		1
Cephalosporin/rifampicin		4	
Flucloxacillin/rifampicin	0	8	1
Flucloxacillin/rifampicin/gentamycin	0		3
Flucloxacillin/tobramycin/gentamycin	0		1
Flucloxacillin/aminoglycoside/rifampicin		4	
Flucloxacillin/aminoglycoside		3	
Vancomycin/amikacin	2		
Vancomycin/ceftriaxone	0		13
Vancomycin/ceftazidime	0		1
Vancomycin/ciprofloxacin	2		3
Vancomycin/cefuroxime	0		5
Vancomycin/cefazolin	0		1
Vancomycin/flucloxacillin	4		0
Vancomycin/gentamycin	2		0
Vancomycin/rifampicin		3	
Other		6	

**Table 3:** Treatment strategies in chronic PJI management in 2024.

	Category	*n* (%)
Approach	One-stage in selected cases	40 (87)
	Always one-stage	1 (2)
	Always two-stage	5 (11)
Spacer type—Hip	Prefabricated	21 (50)
	Functional spacer	5 (1)
	Custom-made spacer	1 (2)
	No spacer (Girdlestone)	11 (26)
	Case-dependent	4 (10)
Spacer type—Knee	Cement molds	32 (78)
	Static spacer	4 (10)
	Prefabricated	2 (5)
	Case dependent	3 (7)
Antibiotic holiday	Standard	3 (7)
	Never	11 (26)
	Case-dependent	28 (67)

**Table 4:** Duration of antibiotic treatment following DAIR procedure, one-stage and two-stage revision surgery.

	DAIR Procedure *n* = 77 (%)	One-Stage *n* = 43 (%)	Two-Stage *n* = 41 (%)
6 weeks	5 (6)	8 (19)	12 (29)
6–12 weeks	-	1 (2)	2 (5)
12 weeks	69 (90)	33 (77)	20 (49)
Culture-dependent	3 (4)	1 (2)	7 (17)

## Data Availability

The raw data supporting the conclusions of this article will be made available by the authors on request.

## Abbreviations

The following abbreviations are used in this manuscript:

ASA	American Society of Anesthesiologists
CNS	Coagulase-negative Staphylococci
DAIR	Debridement, antibiotics, and implant retention
EBJIS	European Bone and Joint Infection Society
GNB	Gram-negative Bacilli (GNB)
ICM	International Consensus Meeting
LROI	Dutch Arthroplasty Register
NINJA	The Northern Infection Network Joint Arthroplasty
NOV	Dutch Orthopaedic Association
PJI	Periprosthetic joint infection
SWAB	Dutch Working Party on Antibiotic Policy
THA	Total hip arthroplasty
TKA	Total knee arthroplasty
WOI	Dutch Orthopaedic Infection Society
